# Alpelisib and Immunotherapy: A Promising Combination for Recurrent and Metastatic Squamous Cell Carcinoma of the Head and Neck

**DOI:** 10.1002/cnr2.70023

**Published:** 2024-10-07

**Authors:** Riham Suleiman, Patrick McGarrah, Binav Baral, Dawn Owen, Jesus Vera Aguilera, Thor R. Halfdanarson, Katharine A. Price, Harry E. Fuentes Bayne

**Affiliations:** ^1^ Division of Medical Oncology Mayo Clinic Rochester Minnesota USA; ^2^ Division of Radiation Oncology Mayo Clinic Rochester Minnesota USA; ^3^ Division of Hematology/Oncology Essentia Health Duluth Minnesota USA

**Keywords:** alpelisib, head and neck squamous cell carcinoma, immunotherapy, PI3K pathway, PIK3CA

## Abstract

**Background:**

Recurrent squamous cell carcinoma (SCC) of the head and neck (SCCHN) remains a formidable clinical challenge despite available treatments. The phosphatidylinositol 3‐kinase (PI3K) pathway has been identified as a potential therapeutic target, and alpelisib, a selective PI3Kα inhibitor, has demonstrated efficacy in certain malignancies. Combining this targeted therapy with immunotherapy has been suggested in previous studies as a promising strategy to bolster the immune response against cancer.

**Cases:**

A 69‐year‐old woman with locoregional recurrence of PIK3CA‐mutated SCC of the left maxilla and cervical nodal metastases. Several chemotherapeutic regimens, including cisplatin, docetaxel, 5FU, chemoradiotherapy, and mono‐immunotherapy, resulted in disease progression. Alpelisib combined with pembrolizumab led to a sustained response for 9 months. A 58‐year‐old man with recurrent metastatic PIK3CA‐mutated SCC of the oropharynx, involving the left lung, hilar, and mediastinal lymph nodes. Despite prior palliative radiation and platinum‐based chemotherapy with pembrolizumab and cetuximab, treatment with alpelisib and nivolumab resulted in a partial response. Severe hyperglycemia and rash led to treatment discontinuation.

**Conclusion:**

Our findings highlight the potential of this innovative therapeutic combination, suggesting a need for further investigations in this setting.

## 1 Introduction

Squamous cell carcinoma (SCC) of the head and neck (SCCHN) is ranked as the seventh most common cancer worldwide, with an estimated annual incidence of approximately 700 000 cases in 2018, resulting in a mortality rate of 350 000 [1]. Despite the availability of various treatment modalities, recurrence is frequently observed in locally advanced SCCHN. This recurrence often leads to significant morbidity and mortality in patients who are not eligible for curative therapy [2, 3].

The treatment landscape for recurrent and metastatic (R/M) SCCHN has been transformed by the advent of anti‐programmed death 1 (PD‐1) immunotherapy. PD‐1 inhibitors, either as monotherapy or in combination with platinum‐based chemotherapy, have demonstrated superior outcomes compared with the erstwhile standard treatment of platinum‐based chemotherapy with cetuximab (EXTREME regimen) [4, 5, 6, 7, 8, 9]. As a result, immunotherapy‐based regimens are the frontline treatment for R/M SCCHN.

Yet, challenges loom, especially when patients progress on immunotherapy, or their disease remains unresponsive to PD‐1 inhibition. These challenges may indicate innate, adapted, or rapidly acquired resistance to immunotherapy. In an attempt to circumvent resistance linked with checkpoint inhibitors such as PD‐1, various strategies have emerged, including the addition of cytotoxic T‐lymphocyte antigen 4 (CTLA‐4) inhibitors [10] and cetuximab [11]. Nonetheless, a significant gap remains in our arsenal of effective treatments in the second‐line setting and beyond and in the identification of predictive biomarkers.

With the strides in genetic sequencing, it has been unveiled that nearly 30% of SCCHN cases manifest PI3KCA amplifications and mutations [12]. Inhibitors of the mammalian target of rapamycin (mTOR) pathway are gaining traction for their potential to recalibrate the immune response, a trait evident in various animal cancer models, including melanoma, lung cancer, thymoma, and breast cancer [13]. Presently, clinical trials are delving into the therapeutic promise of phosphatidylinositol 3‐kinase (PI3K)‐targeted inhibitors, both as monotherapy and in combination regimens for SCCHN.

In this case series, we describe our experiences with an innovative combination treatment of alpelisib and immunotherapy in two patients with R/M PIK3CA‐mutated SCCHN. The rapid and profound response observed in these patients underscores the potential efficacy and promise of this therapeutic combination.

## 2 Cases Presentation

### 2.1 Case 1

A 69‐year‐old woman presented to the Mayo Clinic in July 2022 with locoregional recurrence a decade after undergoing surgical resection for a T3N0M0 SCC of the left maxilla. A staging PET‐CT scan revealed a significant fluorodeoxyglucose (FDG)‐avid left sinonasal mass with peri‐neural spread. Additionally, multiple FDG‐avid ipsilateral sub‐centimeter cervical lymph nodes were identified, raising suspicion of nodal metastasis; however, there was no evidence of distant disease. Given the bulky and symptomatic nature of the locoregional recurrence, the decision was made to initiate induction chemotherapy using a 3‐drug regimen (docetaxel 67.5 mg/m^2^ on Day 1, cisplatin 67.5 mg/m^2^ on Day 1, and 5‐fluorouracil (5‐FU) 675 mg/m^2^ on Days 1–5), which would then be followed by concurrent chemoradiotherapy (CRT) with 45 mg/m^2^ weekly cisplatin.

Three months post‐CRT, a PET‐CT scan revealed persistent disease in the maxillary sinus and a new suspicious nodule in the right lung. A biopsy confirmed residual SCC with a combined positive score (CPS) of 15%. Next‐generation sequencing (NGS) using Tempus‐xT assay (Chicago, IL), including targeted NGS of 648 oncogenes, identified two actionable mutations: a PIK3CA c. 3140A > T p.H1047L missense gain‐of‐function mutation (34.4%) and an epidermal growth factor receptor (EGFR) p.N771delinsGF exon 20 in‐frame insertion gain‐of‐function mutation (18.3%). Considering the low disease burden, the asymptomatic nature of the disease, and a CPS of 15%, pembrolizumab monotherapy was initiated.

**FIGURE 1:**
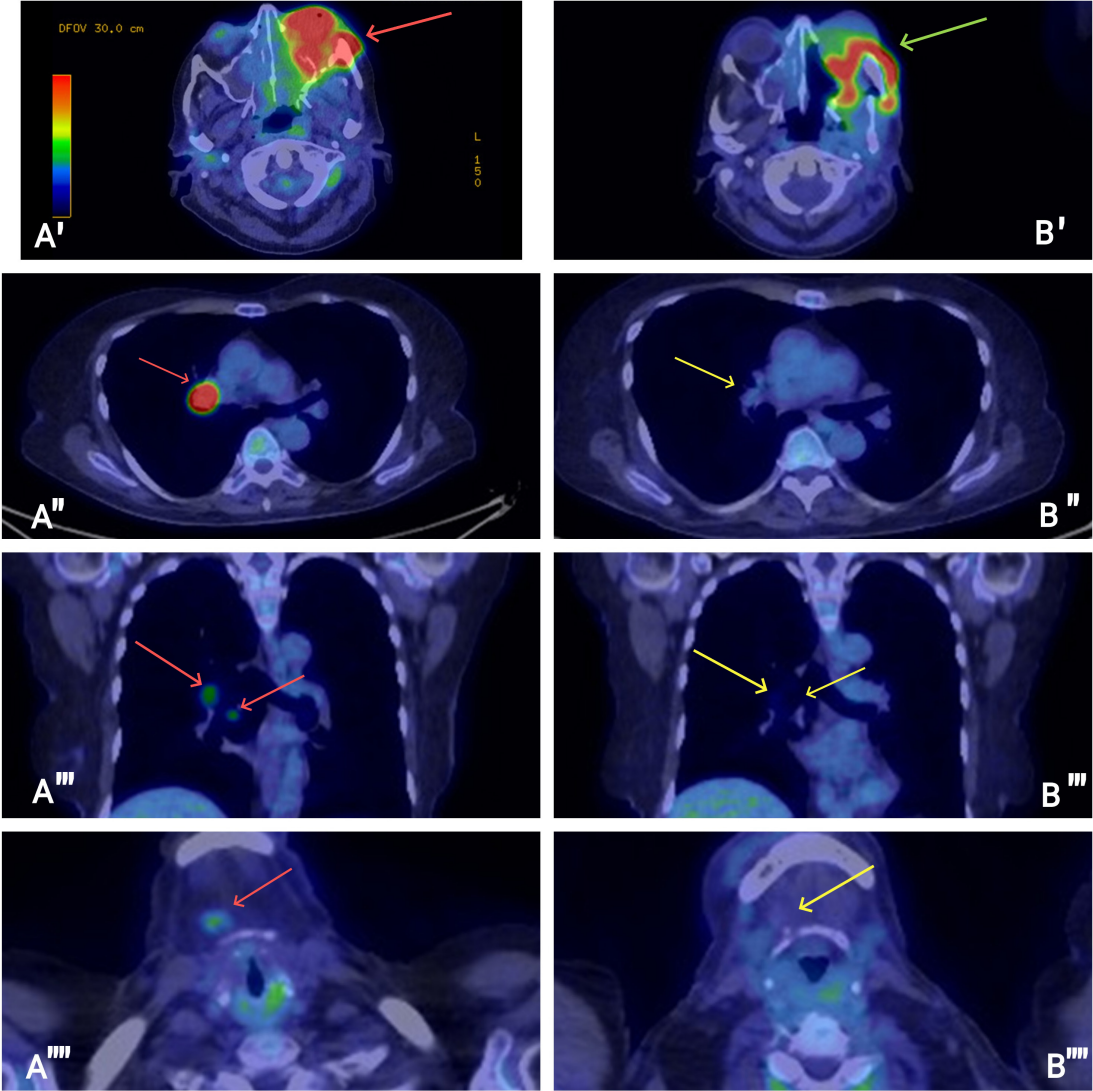
(A) PET/CT scan illustrating disease progression in the left maxilla, right hilum, right lung, and cervical lymph nodes before the commencement of alpelisib and immunotherapy (red arrows). (B) PET/CT scan captured 6 months post‐initiation of alpelisib and immunotherapy, demonstrating a significant response in the left maxillary region (green arrow) and a complete metabolic response in the right lung and previously involved lymph nodes (yellow arrows).

Three months into immunotherapy, a restaging PET‐CT scan revealed disease progression in the maxillary area, right lung, and new hilar lymphadenopathy. Clinically, the patient exhibited a protruding and painful tumor from the left maxillary sinus. Consequently, an NGS‐guided approach was adopted, and alpelisib 300 mg once daily was combined with pembrolizumab 200 mg every 3 weeks. This regimen resulted in a significant response in the maxillary sinus and a complete metabolic response in both the lung and the previously involved lymph nodes as seen in the restaging PET‐CT scan 3 months after initiating this therapy (Figure 1 **)**. During alpelisib treatment, the patient experienced Grade 3 hyperglycemia, which was successfully managed with inpatient, followed by outpatient insulin management.

After 9 months of sustained response, cancer progression was observed in the liver, face, and right hilar areas. The subsequent therapeutic strategy involved a combination of capecitabine 1000 mg/m^2^ twice daily on days 1–14 and pembrolizumab 200 mg every 3 weeks. However, pembrolizumab had to be discontinued due to Grade 3 immune‐related hepatitis. A follow‐up PET scan a month later showed advanced disease progression, with numerous FDG‐avid metastatic lesions in the pulmonary, nodal, skeletal, and hepatic regions. As a result, treatment with cetuximab 400 mg/m^2^ loading dose followed by 250 mg/m^2^ weekly and paclitaxel 80 mg/m^2^ once weekly for 3 weeks was initiated, leading to a partial response. However, due to worsening fatigue, the patient chose to cease all treatments and entered hospice care.

### 2.2 Case 2

A 58‐year‐old man was referred to our institution in September 2019 for the management of R/M HPV‐associated SCC of the oropharynx. Two years post‐initial treatment, which comprised of resection of a T4N2M0 tumor followed by adjuvant radiation, a lesion was identified in the upper lobe of the left lung. A biopsy of this lesion revealed SCC positive for p16, CK5, and p40, with a CPS score of 20%. Although there was rare weak nonspecific staining for TTF1, the strong positivity for p16 supported a metastasis from the patient's known HPV‐positive oropharyngeal SCC. Subsequently, the patient underwent a wedge resection of the left upper lobe lesion and thoracic lymph node dissection. Pathological assessment showed lymphovascular invasion and focal invasion of the visceral pleura, with no nodal involvement. NGS analysis using Tempus‐xT assay (Chicago, IL), detected actionable mutations in the following genes: PIK3CA c.1624G > A p.E542K missense exon 9 gain‐of‐function mutation (24.3%), PIK3CA c.2176G > A p.E726K missense exon 13 gain‐of‐function mutation (12.3%), and a copy number loss in BRCA1‐associated ring domain protein 1 (BARD1).

A month following the confirmation of metastatic disease, the patient underwent a second thoracotomy and lymph node dissection due to the emergence of two new lung lesions. Restaging with a PET‐CT scan 7 months post‐resection revealed disease progression in the oropharynx, prompting a short course of palliative radiation. Subsequent scans identified new disease areas in the left lung, accompanied by hilar and mediastinal lymph node involvement. As a result, the patient commenced treatment with cisplatin 80 mg/m^2^ every 21 days, 5FU 1000 mg/m^2^ every 21 days, and pembrolizumab 200 mg every 21 days, which led to a partial response on the restaging PET‐CT scan 3 months later. However, 10 months into this regimen, the patient developed brachial plexopathy attributed to disease progression of the mass at the left lung apex. Further restaging scans highlighted disease progression in the mediastinal lymph nodes. Consequently, the patient transitioned to a regimen of carboplatin (AUC) 5 mg/mL/min, paclitaxel 175 mg/m^2^, and weekly 250 mg/m^2^ cetuximab. After six cycles, a partial response was observed on restaging scans, but the treatment had to be paused for 2 months due to an active COVID‐19 infection.

After the interruption of therapy, his cancer‐related plexopathy intensified, and interval scans indicated further progression of the left lung mass. Subsequently, the patient was referred to our institution for alternative therapeutic considerations. Several treatment options, including the resumption of chemoimmunotherapy, were presented to the patient. However, he expressed a marked inclination for a nonchemotherapy‐based approach. Our radiation oncology team evaluated the possibility of SBRT, but due to the patient's extensive prior radiation and the metastases' proximity to critical structures, systemic therapy, and close surveillance were prioritized. Considering the patient's preferences, NGS results, and the radiation oncology evaluation, we decided to commence treatment using alpelisib 300 mg once daily in conjunction with nivolumab 240 mg every 2 weeks.

**FIGURE 2:**
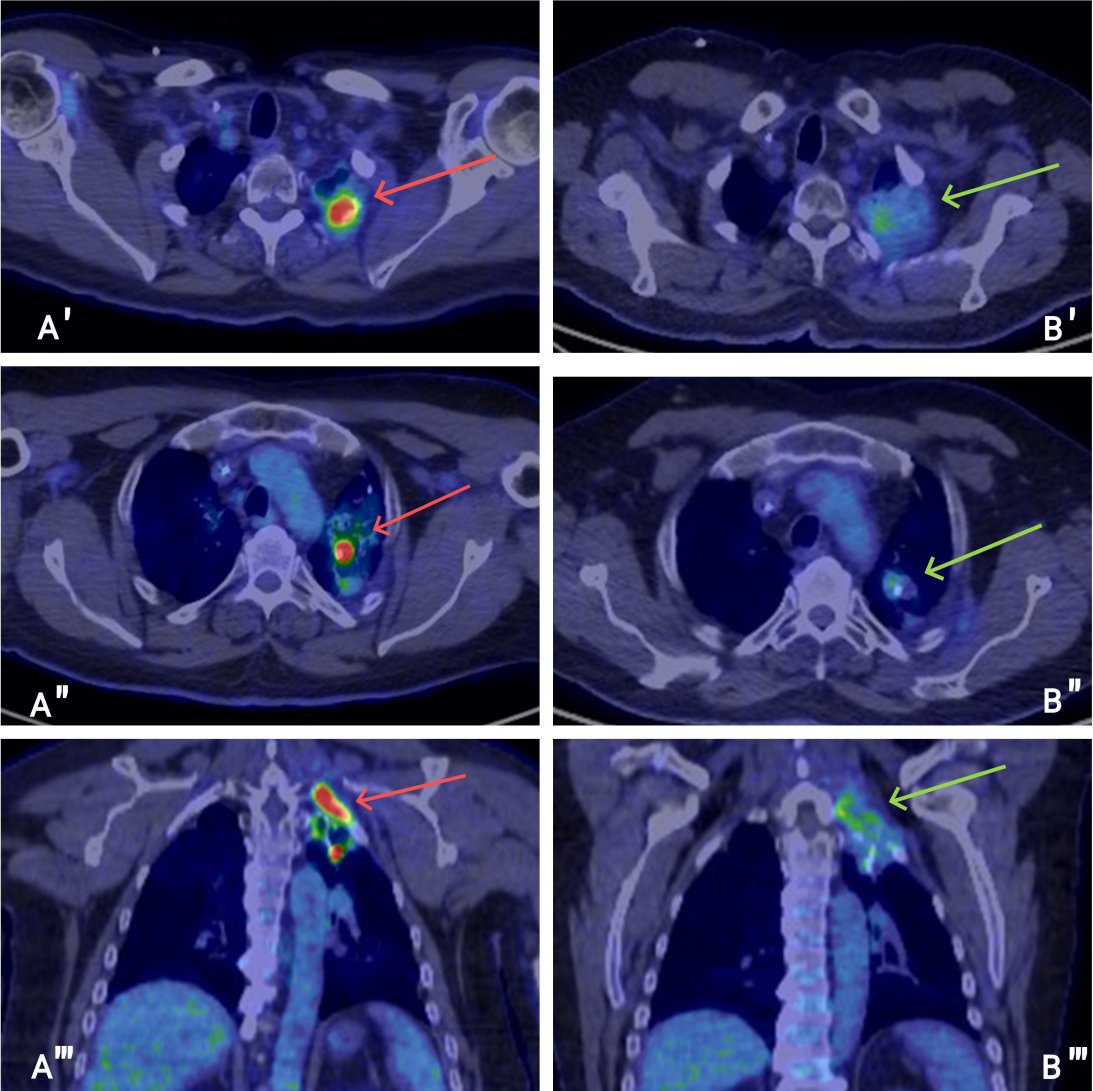
(A) PET/CT scan depicting disease progression in the apex of the left lung before initiating treatment with alpelisib and immunotherapy (red arrows). (B) PET/CT scan taken 1 month post‐initiation of alpelisib and immunotherapy, showcasing an outstanding partial response in the apex of the left lung (green arrows).

Soon after beginning this regimen, the patient reported a swift alleviation of his plexopathy symptoms. A follow‐up PET‐CT scan after 2 months revealed a partial response in the left apical mass and no signs of additional disease (Figure 2). However, the patient soon faced complications of Grade 3 hyperglycemia and a Grade 1 rash. Despite initiating intensive antihyperglycemic treatment, including the use of insulin and oral medications, the patient's fasting glucose levels remained significantly elevated necessitating the discontinuation of alpelisib. At this juncture, consolidation CRT was chosen as the next course of action, followed by active surveillance. This decision was based on the observed reduction in tumor size with the novel combination therapy, which decreases radiation exposure to critical structures, the lack of effective alternatives, and the patient's preference for more definitive therapy. Nine months of this consolidation therapy, restaging scans have shown no evidence of disease recurrence.

## 3 Discussion

In this case series, the novel combination of immunotherapy and alpelisib demonstrated a swift and enduring response in two patients with heavily pretreated R/M SCCHN. Such results are promising and warrant further prospective investigation in the setting of a clinical trial.

R/M SCCHN poses a significant management challenge. While first‐line agents are well‐defined, favoring immunotherapy with or without chemotherapy regimens [4], the management landscape becomes less clear for patients experiencing relapse. Salvage therapy with curative intent, such as surgery or reirradiation, may be viable for select cases. However, the majority of recurrent patients ultimately necessitate palliative systemic therapy. The optimal regimen for this subgroup remains undefined, and the choice of subsequent therapy, particularly in the context of relapse on immunotherapy, is subject to ongoing evolution. Despite the presence of current alternatives such as single‐agent cetuximab, or cetuximab in combination with chemotherapy [14], paclitaxel, docetaxel, capecitabine, methotrexate, and afatinib, the lack of consensus on a standard of care and diverse expert opinions underscore the imperative for continuous investigations. Consequently, the role of precision medicine, with the aim to unveil the most appropriate targeted agents for patients facing relapse, emerges as a fitting avenue in this dynamic scenario.

The genomic landscape of SCCHN has revealed numerous somatic mutations, including loss‐of‐function TP53 mutations, CDKN2A deletions, and activating mutations in HRAS or PIK3CA alterations [12]. This landscape further diversifies based on HPV status. For instance, TP53 mutations predominantly affect HPV‐negative tumors, while HPV‐associated cancers frequently exhibit activation in the phosphoinositide 3‐kinase (PI3K) pathway as a primary oncogenic driver [15]. The PI3K pathway is found to be activated in more than 90% of SCCHN [16, 17], including both HPV‐positive and HPV‐negative subsets [15]. Remarkably, between 15% and 30% of SCCHN cases are linked with constitutive activation of the PI3KCA gene, marking it as the most frequently encountered mutation in this pathway [18] accounting for 37% of HPV‐positive SCCHN cases and 27% of HPV‐negative SCCHN cases [18].

The oncogenic PI3K/AKT pathway activation encompasses a myriad of regulatory signals and downstream effectors. Dysregulation of the PI3K pathway can arise from various molecular alterations. These include gain‐of‐function mutations in PI3KCA or INPP4B, amplifications of PI3KCA or AKT, and deletions in PTEN or TSC1/2 tumor suppressor genes. Additionally, the overexpression of the upstream EGFR and its cross‐interaction with RAS gain‐of‐function mutations are known contributors to PI3K pathway activation [19]. This pathway also plays a significant role in the epithelial‐mesenchymal transition, a pivotal process in tumor progression and invasion [20]. Moreover, aberrations in the PI3K pathway are not limited to cancer onset; they also challenge the efficacy of primary SCCHN treatments, including resistance to platinum‐based chemotherapy [21], cetuximab [22], immunotherapy, and radiotherapy [23].

Genomic profiling studies and preclinical models have spurred significant efforts to develop therapeutic applications targeting the PI3K pathway for SCCHN. Most PI3K inhibitors under development share a mechanism of action: they competitively interact with the ATP‐binding pocket of PI3K, inhibiting its kinase activity and thereby its oncogenic potential [24]. However, these inhibitors vary in their selectivity. They range from Class I pan‐PI3K inhibitors (e.g., buparlisib, copanlisib, and sonolisib) to isoform‐specific Class I PI3K inhibitors (e.g., idelalisib, alpelisib, and pilaralisib) and even dual mTOR/PI3K inhibitors such as SF1126 [25]. Notably, among these PI3K inhibitors, copanlisib (for follicular lymphoma), alpelisib (for breast cancer and PIK3CA‐related overgrowth spectrum [PROS]), idelalisib, duvelisib (for chronic lymphocytic leukemia [CLL]), and umbralisib (for follicular and marginal zone lymphoma) have been approved by the Food and Drug Administration (FDA), although approvals/accelerated application indications of partial inhibitors (idelalisib, duvelisib, and umbralisib) have been withdrawn [26].

Many clinical trials have explored PI3K‐targeted therapies for SCCHN, either as monotherapy or in combination. Studies examining the efficacy of Class I pan‐PI3K inhibitors in SCCHN have yielded conflicting results. While Phase II clinical trials tested sonolisib (PX‐866) in patients with locally advanced, recurrent, or metastatic SCCHN, either by combining it with docetaxel (NCT01204099) or cetuximab (NCT01252628) showed limited efficacy, the BERIL‐I Phase II trial showed an improvement in progression‐free survival after treatment with Class I pan‐PI3K inhibitor buparlisib (BKM120) plus paclitaxel in patients with SCCHN previously treated with platinum [27].

However, selective isoform inhibitors such as alpelisib have demonstrated encouraging results. For instance, a Phase Ib study combining alpelisib with cetuximab and concurrent radiation therapy for Stage III to IVB SCCHN patients showed a complete response across all participants, with a notable 90% remaining disease‐free over 2 years [28]. Furthermore, a Phase I trial pairing alpelisib with cisplatin‐based CRT for locoregionally advanced SCCHN reported a promising 3‐year overall survival rate of 77.8%, complemented by a manageable safety profile [29]. To provide a detailed overview of the current literature on alpelisib in R/M HNSCC, we have created Table 1 summarizing clinical trials that have investigated its use, both alone and in combination with other agents, for managing R/M HNSCC.

**TABLE 1:** Summary of the cases and clinical trials utilizing alpelisib in recurrent/metastatic head and neck squamous cell carcinoma.

Authors	Title	Journal	Years
Keam, B., M. H. Hong, et al. [30]	Personalized biomarker‐based umbrella trial for patients with recurrent or metastatic HNSCC	*Journal of Clinical Oncology*	2024
Ye, Y., Z. Huang, et al. [31]	Synergistic therapeutic potential of alpelisib in cancers (excluding breast cancer)	*Biomedicine & Pharmacotherapy*	2023
Smith, A. E., S. Chan, et al. [32]	Tipifarnib potentiates the antitumor effects of PI3Kalpha inhibition in PIK3CA‐ and HRAS‐dysregulated HNSCC	*Cancer Research*	2023
Lee, M. J. and H. F. Kao [33]	Promising response with PI3K inhibitor for a patient with heavily pretreated PIK3CA mutation HNSCC	*Journal of Cancer Research and Practice*	2023
Juan, A., C. Segrelles, et al. [34]	Anti‐EGFR conjugated nanoparticles to deliver alpelisib as targeted therapy for head and neck cancer	*Cancer Nanotechnology*	2023
Kessler, L., S. Malik, et al. [35]	Potential of farnesyl transferase inhibitors in combination regimens in squamous cell carcinomas	*Cancers*	2021
Jin, N., B. Keam, et al. [36]	Therapeutic implications of activating noncanonical PIK3CA mutations in HNSCC	*Journal of Clinical Investigation*	2021
Holzhauser, S., N. Wild, et al. [37]	Targeted therapy with PI3K and FGFR inhibitors on human papillomavirus positive and negative tonsillar and base of tongue cancer lines	*Frontiers in Oncology*	2021
Ruicci, K. M., J. Meens, et al. [38]	TAM family receptors in conjunction with MAPK signaling are involved in acquired resistance to PI3Kalpha inhibition in HNSCC	*Journal of Experimental & Clinical Cancer Research*	2020
Massacesi, C., E. Di Tomaso, et al. [39]	PI3K inhibitors as new cancer therapeutics: implications for clinical trial	*OncoTargets and therapy*	2016

The resistance of PI3K pathway alterations‐harboring SCCHN to immunotherapy is shaped not only by the genetic activation of this pathway but also by its broader implications on the cancer‐fighting capacity of the immune system. Specifically, upregulating Class I PI3K genes detrimentally affects both innate and adaptive immunity [40]. This upregulation correlates with the PD‐1 pathway, given its association with heightened PD‐L1 expression in tumor cells [41].

Tim‐3, an inhibitory molecule on T cells, plays a role in inflammation and cancer development [42]. Blocking PD‐1 boosts Tim‐3 expression, indicative of dysfunctional T cells [43]. In SCCHN, PD‐1 hiTim‐3 T cells appear in 30%–50% of cases, potentially explaining the resistance to anti‐PD‐1 therapies [44]. The PI3K pathway, intriguingly, promotes Tim‐3 expression, influencing tumor responsiveness to immunotherapies [44]. This pathway also establishes a suppressive immune environment in myeloid cells by recruiting Ccr‐2‐expressing cells [45], thereby hampering CD8 T cell infiltration, promoting tumor growth, and leading to resistance against immune checkpoint therapies [45].

These mechanisms underscore the potential of PI3K mutations as markers for suboptimal responses to immunotherapies. They advocate for concurrent mutation‐targeted and immunotherapy treatments to enhance SCCHN patient outcomes [46, 47]. Preclinical studies integrating PI3K inhibition with immunotherapy have shown promise: better tumor growth control [48, 49], increased persistence of CD8+ memory T cells [50], and diminished PD‐L1 expression on tumor cells [51]. Multiple Phase I/II clinical trials are currently evaluating this treatment combination in solid tumors such as (NCT02646748) and (NCT02637531). It is worth mentioning that a clinical trial on this combination in SCCHN (NCT04193293) was initiated previously. However, it was terminated due to low enrollment.

In both cases we presented, immunotherapy was used post progression. This approach aligns with the current evidence supporting the continued use of these agents beyond progression to reinvigorate the immune response and bypass resistance mechanisms [52]. Both primary and secondary resistance to immunotherapy can be influenced by tumor intrinsic and extrinsic factors. Studies have shown that combining immunotherapy with treatments like chemotherapy or targeted therapy can overcome resistance, as seen in the Morpheus study (NCT03337698) and the Salous et al. Phase 2 study [53]. The ongoing INSIGNA Phase 3 trial (NCT03793179) will further assess this theory. Our approach suggests that retreatment and combination therapies, such as pembrolizumab with alpelisib, can target diverse tumor clones and overcome resistance, including those caused by PIK3CA mutations.

In clinical trials exploring immuno‐targeted therapy combinations, vigilance for potential side effects is paramount. Although alpelisib generally has a more favorable safety profile compared to pan‐PI3K inhibitors, side effects, especially hyperglycemia, are not rare. Hyperglycemia is reported in approximately 65% of patients, with severe documented instances [54]. This frequency necessitates evaluating fasting plasma glucose (FPG) and HbA1c levels before initiating alpelisib and mandates weekly FPG monitoring during treatment [55]. Mild to moderate hyperglycemia (Grades 1 and 2) can typically be managed with antihyperglycemic drugs, while severe cases (Grades 3 and 4) might require halting alpelisib temporarily [56]. In the cases we detailed, both patients developed hyperglycemia due to alpelisib. Though the hyperglycemia in our first case would typically mandate alpelisib discontinuation based on label guidelines, the exceptional therapeutic response inspired us to persist with alpelisib treatment while aggressively managing the hyperglycemia. Since alpelisib‐related hyperglycemia tends to be dose‐dependent, it may be prudent in the future possible trials of this combination to consider initiating treatment at a lower dosage, trying to ameliorate hyperglycemia development and related complications.

Our case series has several limitations. The single‐patient nature of this report may not be generalizable to a larger population due to individual variations in treatment response. Additionally, the lack of a control group makes it challenging to attribute observed outcomes solely to the combination therapy, as other factors may have influenced the results. The short follow‐up period limits the assessment of treatment durability and potential long‐term side effects. Furthermore, there is a risk of selection bias, as the patient described may represent an exceptional response rather than a typical outcome. Thus, ongoing trials are essential to further support the effectiveness and safety of this combination.

## 4 Conclusion

In conclusion, the significant results from this case series, derived from a regimen merging immunotherapy with PI3K inhibition, highlight the need for more research to validate and build upon these findings. With immunotherapy now incorporated into the first‐line treatment setting, there is a paucity of good therapeutic options in the second‐line setting and beyond. We would advocate for a prospective Phase I/II clinical trial to investigate safety and efficacy in a larger cohort of patients with R/M SCCHN after failure of platinum and immunotherapy.

## Author Contributions

**Riham Suleiman:** conceptualization, investigation, writing – original draft, methodology, writing – review and editing, visualization. **Patrick McGarrah:** writing – review and editing. **Binav Baral:** writing – review and editing. **Dawn Owen:** supervision, resources, validation. **Jesus Vera Aguilera:** writing – review and editing. **Thor R. Halfdanarson:** supervision, writing – review and editing, resources, validation. **Katharine A. Price:** writing – review and editing, supervision, resources, validation. **Harry E. Fuentes Bayne:** funding acquisition, writing – review and editing, supervision, resources, validation, project administration.

## Consent

Written informed consent was obtained from the patient/patient's guardian for the publication of this case series. The present investigation is exempt from IRB review per institutional policy.

## Conflicts of Interest

The authors declare no conflicts of interest.

## Data Availability

The data that support the findings of this study are available on request from the corresponding author. The data are not publicly available due to privacy or ethical restrictions.
